# Advancing Osteoarthritis Research: Insights from Rodent Models and Emerging Trends

**DOI:** 10.26502/josm.511500187

**Published:** 2025-03-14

**Authors:** Resmi Rajalekshmi, Devendra K. Agrawal

**Affiliations:** Department of Translational Research, College of Osteopathic Medicine of the Pacific, Western University of Health Sciences, Pomona, California, USA

**Keywords:** Inflammation, Joint inflammation, Osteoarthritis, Preclinical research, Reactive oxygen species, Rodent models

## Abstract

Osteoarthritis (OA) is a degenerative joint disease that affects millions of individuals worldwide, causing pain, disability, and a significant burden on public health. Preclinical research using animal models is essential to our understanding of the underlying pathogenesis of OA and developing therapeutic strategies. Rodent models, in particular, have become indispensable in studying OA due to their ability to mimic various features of human disease. This review provides an overview of commonly used rodent models of OA, including surgical induction (e.g., destabilization of the medial meniscus and anterior cruciate ligament transection), chemical induction (e.g., monoiodoacetate-induced cartilage degeneration), and genetically modified models. Additionally, age-related OA models that naturally develop OA-like symptoms in aged rodents are also discussed. Despite their utility, rodent models face limitations in fully recapitulating the complexity of human OA. Emerging trends in OA research, including the use of 3D imaging for joint analysis, molecular profiling for deeper insights into disease mechanisms, and advancements in biomarkers for early detection and treatment, are highlighted. These innovations provide new opportunities to refine existing models and enhance the translation of findings to clinical therapies. This critical review provides comprehensive information for researchers working in OA and related fields, promoting a better understanding of the available rodent models and their applications in OA research.

## 1. Introduction

OA is a chronic, degenerative joint disease that primarily affects the cartilage, the smooth tissue covering the ends of bones in a joint. As cartilage deteriorates, bones begin to rub against each other, increasing joint friction and resulting in pain, swelling, stiffness, and reduced mobility [1,2]. OA is also characterized by changes in the subchondral bone and synovial inflammation, contributing to joint stiffness and dysfunction. The disease is the most prevalent joint disorder globally, affecting millions of individuals and representing a leading cause of chronic pain and disability [3]. It commonly impacts the knees, hips, hands, and spine, with symptoms typically worsening over time, gradually limiting mobility and daily function [4].

In 2020, approximately 595 million people globally had osteoarthritis (OA), representing 7.6% of the population, a 132.2% increase since 1990. OA cases are projected to rise by 74.9% for the knee, 48.6% for the hand, 78.6% for the hip, and 95.1% for other types by 2050. Knee OA remains the most prevalent, with elevated body mass index (BMI) contributing to 20.4% of cases. The global age-standardized rate of years lived with disability (YLDs) for OA was 255.0 per 100,000 in 2020, a 9.5% increase since 1990. High-income countries, particularly the U.S., showed the highest rates of OA incidence, prevalence, and YLDs in 2019. That year, 32.97 million OA cases were reported in individuals aged 30 to 44 years, with knee OA being the main contributor. The rise in OA cases has been most significant in high and high-middle socio-demographic index countries, with BMI being the leading risk factor. However, modifiable risk factors like injury prevention and occupational hazards remain underexplored in Global Burden of Disease modeling [5–7].

The etiology of OA is multifactorial, involving both genetic and environmental factors. Genetic predisposition plays a significant role in the development of OA, with several susceptibility loci identified in genome-wide association studies (GWAS) [8]. Specific genes involved in cartilage degradation, inflammatory responses, and extracellular matrix remodeling have been linked to OA, such as those encoding for collagen, aggrecan, and metalloproteinases. However, environmental factors such as obesity, joint injury, and physical activity also contribute to OA development and progression [9–11]. Obesity is a major risk factor for OA, especially in weight-bearing joints such as the knee and hip, where excess body weight increases mechanical stress on the joints. Joint injury, including trauma and repetitive use, accelerates cartilage degradation and is strongly associated with post-traumatic OA. Inflammatory cytokines, such as interleukin-1β (IL-1β), interleukin-33 (IL-33), tumor necrosis factor-alpha (TNF-α), and interleukin-6 (IL-6), are key players in the pathogenesis of OA, contributing to cartilage breakdown and joint inflammation [12–14]. Additionally, studies indicate that vitamin D deficiency may increase the risk of knee and hip OA, particularly in individuals under 60, as shown by radiographic assessments [15–16].

**Figure 1:**
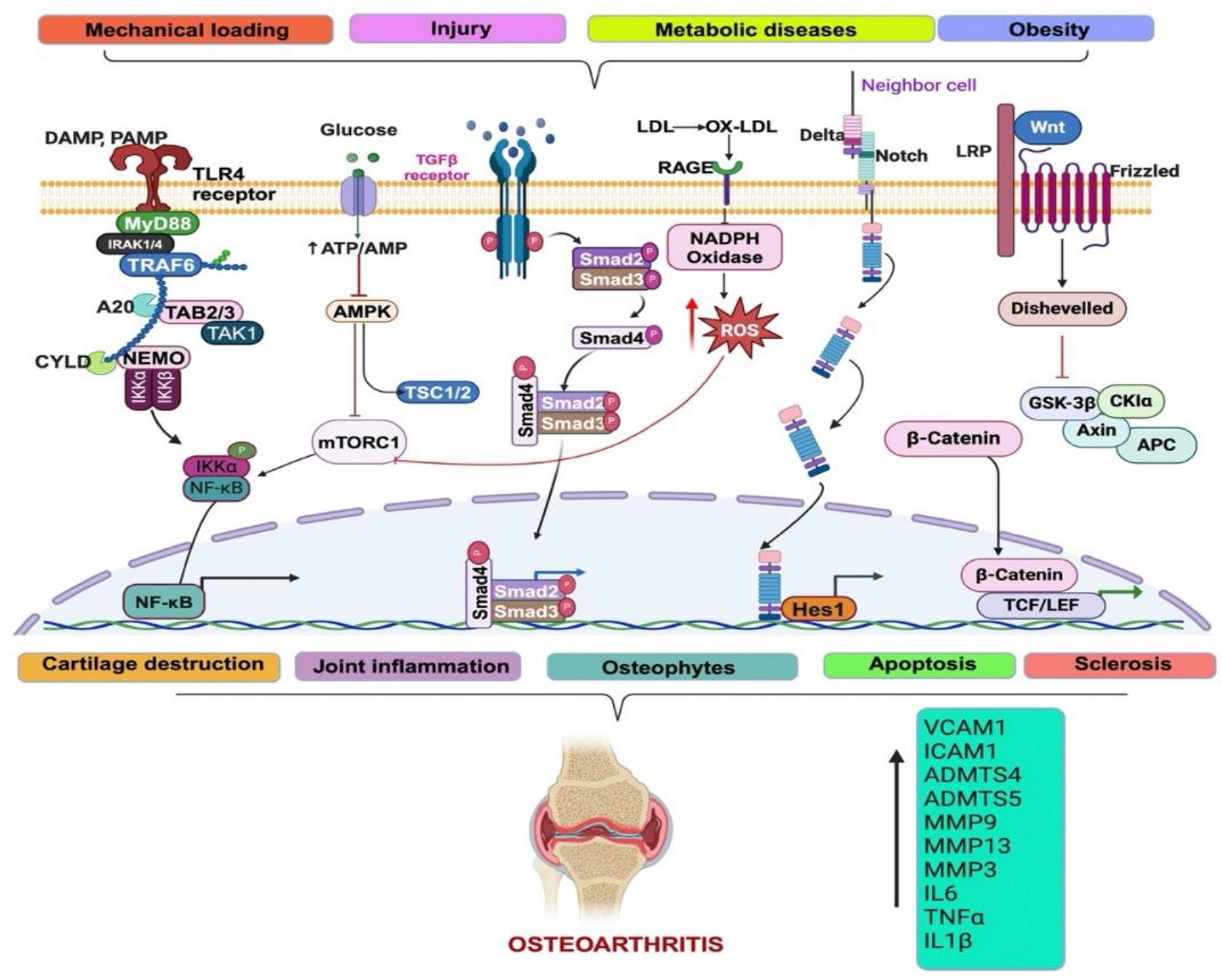
Pathophysiology and Molecular Mechanisms of Osteoarthritis. ADMTS4/5, ADAM metallopeptidase with thrombospondin type 1 motif 4/5; AMPK, adenosine monophosphate (AMP)-activated protein kinase; APC, adenomatous polyposis coli; DAMP, damage associated molecular patterns; Hes1, hairy and enhancer of split-1; ICAM1, intercellular adhesion molecule-1; IL1β, interleukin 1 beta; IL-6, interleukin 6; MMPs, matrix metallopeptidase; mTORC1, mammalian target of rapamycin complex 1; LDL, low-density lipoprotein; MMPs, matrix metallopeptidase; MYD88, myeloid differentiation primary response 88; NADPH, nicotinamide adenine dinucleotide phosphate NEMO, NF-kappa-B essential modulator; NF-κB, nuclear factor-kappa beta; PAMP, pathogen-associated molecular pattern molecules; RAGE, Receptor for Advanced Glycation Endproducts; ROS, reactive oxygen species; SMAD2/3/4, mothers against decapentaplegic homolog; TAK1, transforming growth factor-β-activated kinase 1; TGFβ, transforming growth factor-β; TLRs, toll like receptors; TNFα, tumor necrosis factor alpha; TRAF6, tumor necrosis factor receptor associated factor 6; VCAM1, vascular cell adhesion molecule 1; WNT, wingless-type MMTV integration site.

The pathophysiology of OA (Figure 1) is driven by a complex interplay of mechanical, biochemical, and inflammatory processes that degrade joint integrity. Mechanical stress on joints leads to micro-injuries in the cartilage and subchondral bone, prompting chondrocytes to produce pro-inflammatory cytokines and matrix metalloproteinases (MMPs), such as MMP-13 and ADAMTS-5, which degrade the extracellular matrix [17,18]. This degradation results in cartilage thinning and joint space narrowing. The inflammatory response in OA also involves synovitis, where the synovial membrane becomes inflamed, contributing to pain and stiffness. Synovial inflammation exacerbates cartilage degradation by releasing pro-inflammatory mediators, including cytokines, prostaglandins, and chemokines, which recruit additional inflammatory cells into the joint [19–20]. Furthermore, oxidative stress further exacerbates the disease by damaging cartilage through reactive oxygen species (ROS), which trigger a vicious cycle of cartilage degradation and inflammation. The involvement of oxidative stress has been well-documented, as ROS activate several signaling pathways that promote matrix degradation and chondrocyte apoptosis [21,22].

Recent research has identified several critical signaling pathways involved in OA pathogenesis. One of the key pathways is the Wnt/β-catenin signaling pathway, which regulates cartilage homeostasis and chondrocyte differentiation. Dysregulation of this pathway has been implicated in the development of osteoarthritis, with overactive Wnt signaling leading to cartilage breakdown and osteophyte formation [23,24]. Another important pathway is the transforming growth factor-beta (TGF-β) signaling pathway, which plays a central role in cartilage extracellular matrix production and repair. In osteoarthritis, TGF-β signaling is often impaired, resulting in an imbalance between cartilage breakdown and repair processesAdditionally, DAMPs activate TLRs, RAGE, and NLRP3, triggering NF-κB and MAPK pathways that induce inflammatory cytokines, MMPs, and iNOS, further promoting cartilage destruction [25–28]. The Notch signaling pathway has also emerged as an important regulator of osteoarthritis, with studies showing that activation of Notch signaling in chondrocytes can lead to increased cartilage degradation and inflammation [29,30]. Furthermore, recent evidence suggests that autophagy, a cellular process involved in maintaining cellular homeostasis, plays a critical role in the regulation of osteoarthritis. Impaired autophagy in osteoarthritis contributes to chondrocyte death and matrix degradation.

Animal models are crucial tools in osteoarthritis research, allowing for a controlled investigation of the disease’s pathogenesis. These models replicate various features of human OA, such as cartilage degradation, osteophyte formation, and synovitis. Rodent models, particularly mice and rats, are valuable due to their genetic tractability and the availability of numerous genetically modified strains [31,32]. These models help study the molecular pathways involved in OA, such as the role of inflammatory cytokines, growth factors, and mechanical stress on cartilage homeostasis. Moreover, animal models are essential in preclinical testing of new therapeutic strategies, providing a platform to evaluate the efficacy and safety of potential treatments, including disease-modifying OA drugs (DMOADs), anti-inflammatory agents, and cartilage regeneration therapies. Rodent models are commonly used to test the impact of pharmacological interventions on both structural joint damage and associated pain, bridging the gap between basic research and clinical application.

This review critically reviewed the rodent models of osteoarthritis due to their widespread use in preclinical research. Rodent models are advantageous because of their small size, cost-effectiveness, and genetic similarities to humans. The availability of genetically modified rodent strains allows researchers to study specific genetic and molecular contributions to OA pathogenesis. These models are used to investigate various aspects of OA, including cartilage degradation, subchondral bone changes, and the role of inflammation in disease progression. The review will compare different types of rodent models, including surgically induced models such as anterior cruciate ligament transection (ACLT), chemically induced models using agents like monosodium iodoacetate (MIA), and genetically modified models that mimic specific aspects of human OA. Each model has its strengths and limitations in replicating human OA, with surgically induced models often used to simulate post-traumatic OA, while chemically induced models provide insights into pain mechanisms. Understanding the relevance and applicability of these models is critical for selecting the appropriate one for specific research questions.

## 2. Types of Rodent Models

**Figure 2:**
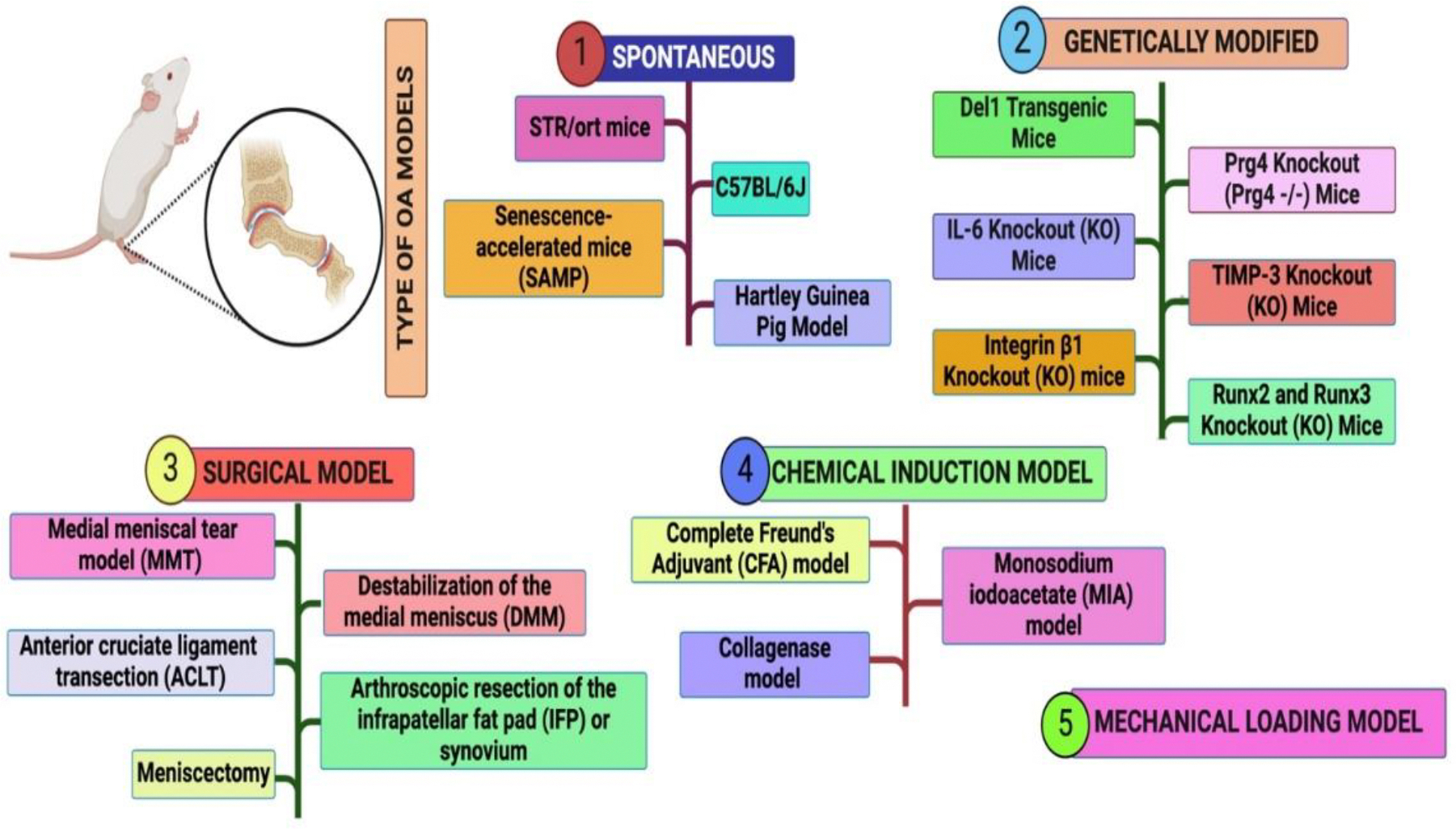
Type of rodent model used to induce osteoarthritis (OA). Del1, developmental endothelial locus-1; Prg4, proteoglycan 4; RunX2/3, runt-related transcription factors; TIMP-3, tissue inhibitor of metalloproteinases-3.

OA can be studied using a variety of rodent models, each chosen based on specific research objectives. Spontaneous models, such as naturally occurring OA (Figure 2 label 1) or genetically modified mice (Figure 2 label 2), are valuable for studying the natural progression and genetic factors of the disease. Surgically induced models (Figure 2 label 3), including meniscectomy or anterior cruciate ligament transection, are commonly used to mimic joint instability and post-traumatic OA. Chemically induced models (Figure 2 label 4), often employed in pain studies, involve the injection of substances like monosodium iodoacetate (MIA) to rapidly induce OA-like changes. Noninvasive mechanical loading models (Figure 2 label 5), such as repetitive mild loading or single-impact injury, are utilized to study the effects of mechanical stress on joint degeneration. The choice of model depends on the study’s focus, whether it is OA pathophysiology, disease progression, therapeutic testing, or pain research. For instance, post-traumatic OA studies frequently use surgical or noninvasive methods, with medial meniscus destabilization (via transection of the medial meniscotibial ligament) being one of the most common approaches to induce instability-driven OA. Each model offers unique advantages and limitations, enabling researchers to address specific aspects of OA.

### 2.1 Spontaneous model

Several mouse strains like C57BL/6, Senescence-accelerated mice (SAM) and STR/ort mice develop OA as they age, providing evidence that genetic predisposition or susceptibility plays a significant role in the disease’s onset. However, the incidence and severity of OA vary across these strains.

#### 2.1.1 STR/ort mice

The STR/ort mouse, an inbred strain with high susceptibility to spontaneous OA, has become a widely recognized model for studying early-onset OA. The STR/ort mouse exhibits key features of human OA, including proteoglycan loss, fibrillation of articular cartilage, degradation of the extracellular matrix, osteophyte formation, and subchondral sclerosis [33,34]. Histological and microcomputed tomography (microCT) analyses have demonstrated that by 40–50 weeks of age, a significant proportion of STR/ort mice exhibit cartilage loss, bone cavitation, erosion, and condylar shape changes in the temporomandibular joint (TMJ) [35]. Additionally, a study investigating the correlation between cartilage histology and subchondral bone parameters revealed significant alterations in cartilage structure and increased expression of OA markers, including aggrecan fragments, MMP-13, and COL10A1, reinforcing the model’s utility in studying OA progression and treatment [36].

#### 2.1.2 C57BL/6J

C57BL/6J mice are another widely used rodent model for age-related joint degeneration and OA. In C57BL/6J mice, age-related changes in knee joint articular cartilage, serum biomarkers (such as CTX-II), and walking speed have been examined. Histological evaluations have revealed progressive cartilage degeneration, with significant increases in OARSI scores from 1 to 6 months of age. Notably, walking speed decreased significantly by 9 months, and serum CTX-II levels peaked at 1 month, indicating a potential early marker for cartilage remodeling [37].

A comparative study assessing OA severity in C57BL/6J mice using the OARSI and ACS grading systems found that aged mice exhibited higher OA severity scores than mice subjected to destabilization of the medial meniscus (DMM) surgery, further establishing C57BL/6J as a relevant model for spontaneous OA and grading system validation [38].

#### 2.1.3 Senescence-accelerated mice

Senescence-accelerated mice (SAM) consist of two sublines: the SAM-prone (SAMP) and SAM-resistant (SAMR), with the SAMP8 strain being particularly notable for its accelerated aging phenotype. These mice are predisposed to age-related conditions such as neurodegenerative diseases, cognitive decline, and sarcopenia, and thus provide a model for studying age-associated OA [39,40].

A study comparing OA-related changes in SAMP8 mice demonstrated that these mice develop OA-like changes earlier than SAMR1 controls, with severe cartilage defects and synovitis observed by 33 weeks of age. Subchondral bone sclerosis and meniscus degeneration were noted even before cartilage degradation, suggesting that SAMP8 mice offer an excellent model for studying primary OA pathogenesis [41].

Another study on the impact of exercise in young SAMP8 mice demonstrated that while low-intensity exercise improved some OA symptoms, it did not prevent cartilage degeneration, emphasizing the role of mechanical stress in OA progression [42].

### 2.2 Hartley guinea pig model

The Hartley guinea pig is a naturally occurring model for moderate to advanced OA, characterized by cartilage loss, proteoglycan depletion, osteophyte formation, and subchondral bone changes. A study on the effects of electroacupuncture (EA) on OA symptoms in this model revealed improvements in movement parameters and antioxidant gene expression but did not significantly alter joint histology. This suggests that while EA may alleviate symptoms, it does not modify disease progression, highlighting the importance of symptom management in OA treatment [43].

### 2.3 Genetically modified mouse models

Genetically modified mouse models have been pivotal in understanding the molecular mechanisms driving OA. These models, which include transgenic, knockout (KO), and knock-in (KI) mice, mimic specific genetic mutations or deficiencies that contribute to OA development, thus providing insights into disease pathogenesis.

#### 2.3.1 Del1 transgenic mice

Del1 transgenic mice express a deletion mutation in type II collagen, which results in cartilage erosion and subchondral bone exposure by 6–10 months. The mutation leads to the production of truncated collagen, a key structural protein in cartilage, accelerating the onset of OA [44,45]. Histological analysis of Del1 KO and wild-type mice revealed reduced cartilage thickness in Del1 KO mice, further supporting the role of DEL1 in cartilage biology and its involvement in OA [46].

#### 2.3.2 Prg4 knockout (Prg4 −/−) mice

Lubricin (Prg4), a glycoprotein that reduces joint friction, is critical for maintaining cartilage integrity [47]. Prg4 −/− mice, which lack this protein, exhibit early joint wear and cartilage damage. Studies have shown increased chondrocyte apoptosis and reduced chondrocyte volume fraction in these mice, highlighting lubricin’s protective role against cartilage degradation and apoptosis [48,49].

#### 2.3.3 IL-6 knockout (KO) mice

IL-6 is an inflammatory cytokine thought to play a protective role in OA. IL-6 KO mice exhibit more extensive cartilage loss in aged males compared to wild-type mice, as well as increased subchondral bone sclerosis and matrix deposition in ligaments. These findings suggest that IL-6 may have an anti-inflammatory role in OA, with its absence exacerbating disease progression in aging mice [50].

#### 2.3.4 TIMP-3 knockout (KO) mice

Tissue inhibitor of metalloproteinases (TIMP)-3 plays a key role in regulating matrix metalloproteinases (MMPs), which are responsible for the breakdown of extracellular matrix components in OA. TIMP-3 KO mice develop mild OA with increased type II collagen cleavage and proteoglycan degradation, making them valuable for studying the role of TIMP-3 in cartilage homeostasis and OA pathogenesis [51].

#### 2.3.5 Integrin β1 knockout (KO) mice

Integrin β1 is essential for collagen binding and cartilage integrity. α1β1 integrin KO mice exhibit severe cartilage degradation and glycosaminoglycan loss, accompanied by increased MMP expression and apoptosis in chondrocytes. These mice are useful for investigating the role of integrins in OA, particularly in cartilage homeostasis and matrix degradation [52].

#### 2.3.6 Runx2 and Runx3 knockout (KO) mice

The Runt-related transcription factor (Runx) family, which includes Runx2 and Runx3, is critical for cartilage maintenance and OA progression. Runx3 KO mice develop accelerated OA following surgical induction, with reduced lubricin and aggrecan expression. In contrast, Runx2 heterozygous knockout mice inhibit OA progression, while homozygous knockout exacerbates disease by reducing type II collagen expression [53].

### 2.4 Surgically induced model

Surgical models of OA are employed to induce joint instability and progressive degeneration by transecting ligaments or removing fibrocartilaginous tissues, effectively mimicking post-traumatic human OA. These models, initially developed in guinea pigs and rats using methods like the medial meniscal tear model [54,55]. The severity of OA progression in these models is influenced by the degree of joint destabilization, with the disease developing within weeks, contrasting the decades-long progression in humans [53,57]. Despite the advanced surgical skills required, these models provide high reproducibility in disease progression timelines.

#### 2.4.1 Medial meniscal tear model (MMT)

The medial meniscal tear (MMT) model is widely used in rodent studies to mimic OA by inducing mechanical instability in the knee joint. The procedure typically involves transecting the medial collateral ligament (MCL) and meniscus, destabilizing the joint, and thereby accelerating the disease’s progression [58]. One variation of the MMT model involves transecting only the meniscus without disrupting the MCL. However, in clinical contexts, meniscal tears refer solely to injuries of the meniscus without MCL involvement. In animal models, the joint destabilization caused by the combined transection of both the MCL and meniscus leads to significant meniscal dysfunction and compromised joint stability, accelerating OA development [59].

One such model, the MMT in rats, has been instrumental in exploring the impact of sex on OA progression. Both male and female Lewis rats undergoing MMT surgery exhibit increased cartilage thickness, proteoglycan loss, elevated subchondral bone mineral density, and larger osteophyte volumes compared to sham-operated controls. Notably, the study revealed sex-specific differences: females exhibited larger normalized cartilage volumes, while males showed larger normalized osteophyte volumes. These findings underscore the relevance of considering sex as a factor in OA research, as they highlight distinct differences in disease progression and severity. The MMT rat model thus presents a useful platform for studying OA development and the impact of sex on therapeutic responses [60].

Further investigations using the MMT model in C57BL/6J mice have provided additional insights into pain-related behaviors and the relationship between OA pathology and pain sensitivity. In this model, male mice demonstrated a bi-phasic pattern of mechanical hypersensitivity, with both acute and chronic phases separated by an intermediate remission phase. Females, on the other hand, showed greater mechanical hypersensitivity during the intermediate phase but exhibited similar limb use compared to males. These pain responses were independent of the degree of cartilage damage, suggesting that the MMT model captures the complex pain features of human OA. The model also demonstrated that while both sexes experienced similar osteophyte formation, males showed more severe cartilage damage, reinforcing the utility of the MMT model in studying the multifaceted nature of OA [61].

The rat MMT model has also been extended to assess disease progression at different stages—early (3 weeks), mid (6 weeks), and late (12 weeks)—which provides a more comprehensive understanding of OA pathogenesis. At 3 weeks post-surgery, tibiae showed proteoglycan loss and fibrillation of articular cartilage, while contrast-enhanced μCT revealed initial cartilage thickening, followed by thinning and degradation at later time points. Subchondral bone thickening became significant by 6 weeks, with osteophytes forming as early as 3 weeks. These changes mirrored those observed in human OA, offering a valuable tool for evaluating therapeutic interventions aimed at treating established OA, a critical step for clinical translation given that most patients present with advanced disease [62].

Another variant of the meniscal injury model, the medial meniscus posterior root tear (MMPRT) model, has been developed to further investigate the association between meniscal tears and OA progression, particularly with regard to subchondral bone insufficiency fractures. The MMPRT model, created by sectioning the medial meniscus posterior root in 12-week-old male CL57BL/6J mice, showed OA progression and medial meniscus extrusion after 12 weeks, with similar Osteoarthritis Research Society International (OARSI) scores to the destabilization of the medial meniscus (DMM) model. However, the MMPRT group exhibited more severe subchondral bone changes, including tibial plateau and femoral condyle destruction, which were not seen in the DMM group. These findings suggest that the MMPRT model is a valuable tool for studying the pathological changes associated with meniscal root tears and offers a unique perspective on OA progression, including bone damage that is not typically captured in other models [63].

#### 2.4.2 Meniscectomy

Meniscectomy, the surgical removal of the meniscus, increased the likelihood of developing radiographic OA and significantly heightened knee pain over time [64]. In rodent models, OA was induced through various meniscectomy approaches, including total, partial, unilateral, and bilateral procedures, with partial meniscectomy achieved by unilaterally cutting the medial meniscus.

One study explored the effect of ovariectomy-induced osteoporosis (OP) on OA progression in a rat meniscectomy model, focusing on subchondral bone changes and pain-related behaviors. The rats were divided into four groups: sham, OP, OA, and OP plus OA, with analysis conducted on histological changes, osteoclast activity, subchondral bone microstructure, and pain behaviors. The findings revealed that rats with both OP and OA exhibited increased calcified cartilage and subchondral bone damage, with elevated osteoclast density in the weight-bearing regions. Additionally, the OP plus OA group displayed more porous trabecular bone compared to OA rats, and loss of tidemark integrity was most pronounced in this group. Importantly, there was a positive correlation between subchondral osteoclast density and bone damage scores, highlighting the role of osteoporosis in exacerbating OA progression [65].

Another study utilized a preclinical surgical model of OA involving anterior cruciate ligament transection combined with medial meniscectomy (ACLT+MMx) in Wistar rats. This model successfully induced OA, confirmed by microscopic analysis showing progressive cartilage degeneration over a 12-week period. The study focused on evaluating the serological detection of OA-related biomarkers, with analysis conducted at two timepoints along with blood cell counts, bone electrolytes, and biochemical analysis. The findings revealed significant reductions in total vitamin D3 and C-telopeptide fragments of type II collagen (CTX-II) in chronic OA rats compared to sham-operated controls. Conversely, serum levels of adiponectin, leptin, and matrix metallopeptidase (MMP3) were significantly elevated in the OA rats. Interestingly, no significant changes were observed in inflammatory markers, blood cell composition, or biochemical profiles following surgery [66].

A further investigation into the effects of mechanical loading on OA was conducted using female C57BL/6 mice, where OA was surgically induced by transecting the medial collateral ligament and removing the medial meniscus. Mechanical loading was applied daily for 2 weeks to one group of OA mice (OAL) while the other group remained untreated (OA). Histological analysis showed severe cartilage degradation in the OA group, including proteoglycan loss and hypocellularity, while mechanical loading mitigated these effects. The OAL group also exhibited reduced synovitis and a lower number of MMP13-positive cells compared to the OA group. Additionally, mechanical loading decreased the number of iNOS-positive cells, suggesting a reduction in M1 macrophages, and modulated the PI3K/AKT/NF-κB signaling pathway [67].

#### 2.4.3 Anterior cruciate ligament transection (ACLT)

ACLT injury is a key model for studying OA progression, as it causes cartilage degeneration and joint instability [68]. ACL injuries are strongly linked to posttraumatic osteoarthritis (PTOA), a degenerative joint disorder affecting millions [69]. Factors such as joint damage, inflammation, and altered kinematics contribute to PTOA, with 24.5% to 50% of ACL injuries leading to PTOA within 10 to 20 years, even after reconstruction [70].

Several studies have utilized the ACLT model to explore different aspects of OA progression. In one study, male Sprague-Dawley rats underwent unilateral ACLT, and functional outcomes such as weight-bearing capacity, mechanical allodynia, motor function, and gait were assessed. The rats received weekly intra-articular injections of Dulbecco’s phosphate-buffered saline (DPBS) or a synthetic biomimetic boundary lubricant for three weeks post-surgery. Results indicated functional impairments up to four weeks post-surgery, with moderate histological signs of OA observed at 20 weeks. Additionally, MRI imaging of rat cadaver knees highlighted the importance of optimal intra-articular injection volumes (20–30 μl) to avoid peri-articular leakage, making this model useful for intra-articular therapy studies [71].

The VEGF-A/VEGFR2 signaling pathway has also been studied in the context of ACLT-induced OA. Moderate OA was induced in C57BL/6 mice through ACLT surgery, and histological analyses revealed time-dependent cartilage degeneration, accompanied by increased expression of ADAMTS5 and decreased collagen II levels. Immunofluorescence identified vascular invasion as early as one week post-surgery, with further intensification by 8 and 12 weeks. Western blot analysis confirmed upregulation of key inflammatory markers such as VEGF-A, VEGFR2, COX-2, and iNOS, suggesting a critical role for angiogenesis and inflammation in OA progression. These findings underscore the utility of the ACLT model in exploring molecular pathways associated with OA [72].

The ACLT model has also been utilized to evaluate novel therapeutic strategies for OA in ten-week-old male Sprague Dawley rats. One such study investigated the use of thermo-responsive hyaluronan-based hydrogels combined with human chondroprogenitor cells. This combination showed promise in slowing OA progression, as demonstrated by tomography, histology, and histological scoring analyses. Importantly, no adverse effects were noted, suggesting the biocompatibility of the hydrogel-cell combination in the ACLT-induced OA model [73].

Biomechanical changes following ACL injury have also been studied, offering insights into the relationship between ACL rupture and OA development. A finite element analysis (FEA) study simulated the biomechanical effects of ACL injury and identified abnormal stress distribution in the medial and lateral femoral and tibial cartilage during knee flexion and extension. Pathological examinations of the medial tibial cartilage in ACLT-induced OA C57BL/6 mice showed significant cartilage degeneration at 4 weeks post-surgery, with the degeneration stabilizing thereafter. This study underscores the biomechanical alterations that occur following ACL injury, which contribute to the development of OA [74].

Furthermore, a recent study developed a nonsurgical method to induce isolated ACL tears in C57BL/6 mice, offering a more clinically relevant and cost-effective alternative to the traditional ACLT model. This method successfully mimicked ACL rupture and meniscal injury without affecting other ligaments, making it a promising tool for studying PTOA progression. The manual procedure demonstrated increased osteophyte volume at eight weeks, highlighting its potential as a simple and reproducible model for investigating post-traumatic OA [75].

#### 2.4.4 Destabilization of the medial meniscus (DMM)

The destabilization of the medial meniscus (DMM) surgical model is widely utilized to investigate the progression of OA in rodent studies. This model involves sectioning the medial meniscotibial ligament, which anchors the medial meniscus to the tibial plateau, inducing joint instability that initiates cartilage degradation as early as two weeks post-surgery. The progressive pathological changes observed include subchondral bone sclerosis and osteophyte formation, making the DMM model a robust tool for studying OA pathogenesis [76,77].

Recent research has emphasized the pathological changes in menisci and ligaments during OA development using the DMM model, alongside other murine models such as the Str/ort spontaneous OA model and post-traumatic OA models. Histological analyses of these models have revealed significant alterations in the menisci, including ossification, hyperplasia, cell hypertrophy, increased collagen type II deposition, and Sox9 expression, which correlate with the severity of articular cartilage lesions. Furthermore, extracellular matrix changes, chondrogenesis at the tibial attachment sites of anterior cruciate ligaments, and ossification in collateral ligaments have been documented. Micro-computed tomography (μCT) imaging has confirmed increased mineralized tissue volume in the joint space as OA progresses, highlighting the interplay between soft tissue changes and joint stability in the pathophysiology of OA [78].

In another study, the consistency of the DMM model was evaluated by comparing surgeries performed with and without a stereomicroscope. This study included 70 male C57BL/6 mice assigned to DMM with stereomicroscope, DMM by naked eye, and sham surgery groups. Functional assessments such as weight-bearing, von Frey test, and gait analysis were conducted over 8 to 16 weeks. Although both surgical approaches induced similar reductions in weight-bearing and pain thresholds, the stereomicroscope group demonstrated significantly less variability in these outcomes and in histological OA severity, as measured by the OARSI scoring system. This finding suggests that while DMM surgery aided by a stereomicroscope is more technically demanding, it yields a more homogeneous OA model with reduced variability in pain and histopathological outcomes [79].

In addition to procedural refinements, pharmacological interventions have been evaluated in DMM-induced OA models. For instance, the effects of zoledronic acid (ZOL), a bisphosphonate, were studied in a DMM-induced OA model in rats. Seventy-two male Sprague-Dawley rats were divided into Sham+PBS, DMM+PBS, and DMM+ZOL groups, with ZOL administered at 100 μg/kg twice weekly for four weeks. Histological and μCT analyses revealed that early OA was marked by dominant subchondral bone resorption, followed by increased bone formation in later stages. ZOL effectively suppressed early bone resorption by inhibiting Wnt5a signaling, thereby improving subchondral bone remodeling, reducing cartilage degeneration, and delaying OA progression. These results underscore the potential of targeting osteoclast activity and Wnt5a signaling in therapeutic strategies for OA [80].

Histological and immunohistochemical studies in DMM-induced OA models have also provided valuable insights into early-stage OA development. In one study, thirty-six male rats were divided into control and OA groups, with histological analyses performed at multiple postoperative time points (1, 3, 7, 10, and 14 days). An increase in cellular density and expression of cartilage degradation-associated proteins was observed from the first day post-surgery. By day three, histological changes such as chondrocyte death, reduced matrix staining, and superficial fibrillation were evident, accompanied by a compensatory increase in matrix staining. By day seven, the Osteoarthritis Research Society International score showed thinner cartilage, and further cartilage fissuring and matrix loss were noted by day ten. Proteoglycan 4-positive cell density increased by day seven, reflecting ongoing compensatory changes in cartilage. These findings highlight the progression of early traumatic OA and provide a framework for understanding its onset and progression [81].

Dynamic remodeling of the synovium and infrapatellar fat pad (IFP) in DMM-induced OA has also been studied using lineage-tracing techniques in Col2a1-Cre Rosa-tdTomato, Dpp4-CreER Rosa-tdTomato, and Prg4-CreER Rosa-tdTomato reporter mice. Proliferation of Dpp4+ mesenchymal progenitor cells (MPCs) in the synovium following DMM led to the generation of Prg4+ synovial lining fibroblasts (SLFs) and myofibroblasts (MFs). Adipocyte loss and incomplete IFP regeneration were observed alongside synovial hyperplasia, characterized by persistent Prg4+ SLFs and αSMA+ Scx1+ MFs. Gene expression analysis revealed upregulation of pathways associated with fibrosis, extracellular matrix remodeling, and inflammation, while adipogenesis pathways were suppressed. These findings underscore the critical role of mesenchymal cells in OA progression and highlight the complex interplay between soft tissue remodeling and joint degeneration in rodent models of OA [82].

#### 2.4.5 Arthroscopic resection of the infrapatellar fat pad (IFP) or synovium

The removal of the IFP or synovium is another model used to induce OA in experimental studies. This model aims to replicate the inflammatory processes associated with OA by surgically removing the IFP or synovium, both of which are key contributors to joint homeostasis and inflammation. The procedure leads to alterations in joint mechanics, changes in the synovial fluid composition, and the induction of inflammatory mediators that accelerate cartilage degradation [83]. Histological analysis typically reveals the onset of cartilage damage, subchondral bone changes, and synovial inflammation, mimicking early-stage OA.

A notable study using male Dunkin-Hartley guinea pigs investigated the effects of IFP and associated synovium (IFP/SC) excision on OA progression. In this model, 18 animals underwent unilateral IFP removal at 3 months of age, with the contralateral knee serving as an internal control. The outcomes were evaluated through gait analysis, microcomputed tomography (microCT), histopathology, transcript expression profiling, immunohistochemistry, and biomechanical testing. Despite no significant differences in gait between experimental and control knees, microCT findings demonstrated reduced OA-related changes in the IFP-removed knees, including improved OA scores, increased osteophyte formation, and enhanced trabecular bone mineral density. Histological analyses confirmed the replacement of the IFP with fibrous connective tissue (FCT), while cartilage structure and proteoglycan content were maintained. Furthermore, transcriptomic assessments indicated reduced expression of adipose-related molecules and inflammatory mediators in the FCT-affected knees, suggesting that the excised IFP may contribute to mitigating inflammatory and degenerative processes [84].

### 2.5 Chemically induced models

Chemical models of OA in rodent studies involve the intra-articular administration of chemical agents such as monosodium iodoacetate (MIA) or collagenase to induce joint degeneration resembling human OA [85]. These models simulate the progressive cartilage degradation and inflammation characteristic of OA, allowing researchers to study the disease’s pathophysiology and evaluate potential therapeutics. MIA inhibits glycolysis in chondrocytes, leading to cartilage destruction, while collagenase degrades collagen, mimicking the enzymatic breakdown of the extracellular matrix [86]. Both models are valuable for understanding OA progression and testing the efficacy of interventions targeting joint preservation and pain relief.

#### 2.5.1 Monosodium iodoacetate (MIA) model

MIA is widely used in OA research due to its ability to produce a consistent, rapid onset of pain-like symptoms and OA-like lesions in rodents. MIA works by inhibiting glyceraldehyde-3-phosphate dehydrogenase, disrupting glycolysis, leading to chondrocyte death, cartilage degeneration, and subchondral bone changes, including osteophyte formation [87]. The resulting joint pathology closely mimics human OA, making it a valuable model for evaluating pharmacologic treatments for OA pain. Although MIA induces acute inflammation followed by degenerative cartilage changes and hyperalgesia, the precise mechanisms underlying its effects remain incompletely understood.

The MIA model has been utilized in numerous studies to evaluate the therapeutic potential of various interventions. One such study investigated the efficacy of LI13019F1 (Serratrin^®^), a composition of Boswellia serrata gum resin fractions, in rats with MIA-induced OA. A single 1 mg MIA injection into the right hind knee induced significant joint pain and mechanical hypersensitivity, typical of MIA-induced OA. Treatment with different doses of LI13019F1 (150 and 300 mg/kg) for 28 days resulted in improved pain sensitivity and increased paw withdrawal latency, suggesting the potential of Boswellia serrata compounds in alleviating OA symptoms. Histological analysis further revealed that MIA-treated rats exhibited cartilage erosion and chondrocyte loss, while LI13019F1-treated rats demonstrated improved cartilage integrity, indicating its therapeutic effects on cartilage health in the context of MIA-induced OA [88].

Another investigation focused on the dose-dependent progression of OA in Sprague-Dawley rats, where different MIA doses (0.25 mg to 4.0 mg) were administered over 12 weeks. This study revealed that higher MIA doses (1.0 mg and above) resulted in rapid and severe joint changes visible as early as the first week, whereas lower doses induced more gradual OA progression. This dose-dependent model allows for the study of specific OA stages and facilitates the evaluation of treatments targeting different disease stages [89].

Further research into the relationship between inflammation and subchondral bone remodeling in MIA-induced OA models demonstrated that MIA injection (3 mg/50 μL) in Wistar rats led to moderate cartilage degeneration by week two and severe degeneration by week four. Inflammation was marked by elevated proinflammatory cytokines such as IL-1β and TNF-α, and accelerated subchondral bone remodeling was observed, with decreased bone density and altered morphology. These findings highlight the critical role of inflammation in driving cartilage and bone structural changes in OA [90].

The MIA model has also been used to evaluate the anti-inflammatory effects of treatments like Pudilan Tablets (PDL) and indomethacin in mice, where a 0.75 mg MIA injection induced OA. The MIA group showed significant cartilage damage, confirmed by histology and elevated proinflammatory cytokine levels, which were significantly reduced by PDL and indomethacin, suggesting their potential as anti-inflammatory agents in OA management [91]. Additionally, the efficacy of PL02 in treating OA was assessed in both mice and rats. In a pilot study, Wistar rats were divided into four groups: Sham control, disease control (MIA), positive control (MIA + Indomethacin), and PL02 treatment group. OA was induced via intra-articular MIA injection (0.5 mg/25 μL), and pain, inflammation, and joint degradation were evaluated over 28 days. MIA-treated rats showed elevated TNF-α and IL-1β levels, confirming the model’s success. PL02 treatment exhibited antioxidant, anti-inflammatory, anti-catabolic, and chondroprotective effects, alleviating pain, reducing inflammation, and preventing cartilage and subchondral bone degeneration [92].

Further studies using adult male Sprague-Dawley rats induced OA through a single intra-articular injection of MIA (3 mg/50 μL), leading to chronic inflammation over 20 days. MIA-induced OA rats exhibited significant pain, reduced motor function, and joint swelling, which was confirmed by radiographic imaging showing joint space narrowing, osteophyte formation, and articular surface roughness. Elevated levels of TNF-α, MMP-13, and altered renin-angiotensin-aldosterone system (RAAS) signaling were observed, indicating the inflammatory and oxidative stress associated with OA. Histological examination revealed thinned articular cartilage, subchondral bone thickening, and severe synovitis with fibrosis. Treatment with DIZE, an activator of ACE2, and losartan, an angiotensin receptor blocker (ARB), reversed these degenerative changes, with DIZE showing more significant improvements in restoring joint architecture and reducing inflammation [93].

The MIA model has also been used to assess the efficacy of Ovomet^®^, a natural eggshell membrane supplement, in both mice (1 mg/10 μL) and rats (3 mg/50 μL). MIA-induced OA led to severe cartilage damage and osteophyte formation, while ESM-treated animals showed improved cartilage integrity and reduced pain, particularly in the early stages of OA. Inflammatory markers such as IL-1β and COX-2 were significantly reduced in the ESM-treated groups, demonstrating the potential of this natural supplement in mitigating OA progression by reducing inflammation and cartilage degradation [94].

Finally, the MIA model has been used to explore the analgesic effects of green-light-emitting diodes (GLED) in male and female Sprague-Dawley rats. MIA injections (3 mg MIA in 15 μL saline) caused mechanical hypersensitivity and elevated proinflammatory cytokines, such as TNF-α, IL-1β, and IL-6. GLED exposure significantly alleviated mechanical hypersensitivity in both sexes, with a faster and more pronounced response in males. The analgesic effects were sustained for up to five days post-exposure and were associated with a reduction in proinflammatory cytokines, suggesting that GLED could offer a promising non-invasive therapy for OA pain management [95].

#### 2.5.2 Complete freund’s adjuvant (CFA) model

The CFA model is a widely used preclinical method for inducing OA by injecting CFA, an emulsion containing inactivated *Mycobacterium tuberculosis*, into the knee joint of rodents [96]. This induces synovitis, cartilage degradation, subchondral bone changes, and pain-related behaviors like hyperalgesia and allodynia, mimicking the inflammatory and structural features of OA. The model is simple, reproducible, and effective for studying inflammation-driven OA and testing analgesic or anti-inflammatory therapies.

A study utilizing the CFA model investigated the therapeutic effects of electroacupuncture (EA), swimming (SW), and their combination (EA + SW) on CFA-induced OA in male Wistar rats. In this model, CFA (100 μL) was injected into the ankle joint, leading to significant inflammatory responses, including paw edema, neutrophilic infiltration (evidenced by elevated MPO levels), and nociceptive behaviors such as reduced cold latency scores. Seven days after CFA injection, treatments were initiated and continued for 20 days to evaluate their efficacy in mitigating inflammation and pain. All intervention groups (EA, SW, and EA + SW) demonstrated reductions in inflammation and nociception compared to the untreated CFA group. Among these, the combination therapy (EA + SW) delayed OA progression based on inflammatory parameters, such as reduced paw edema and neutrophilic infiltration [97].

#### 2.5.3 Collagenase model

The collagenase-induced OA model is a commonly used method to study OA pathogenesis and test therapies. In this model, collagenase enzymes, typically type II, are injected into the joint, causing collagen degradation in cartilage and subchondral bone, which mimics OA’s cartilage breakdown and inflammation [98]. Its simplicity, reproducibility, and rapid induction of OA-like changes make it ideal for studying early OA [99]. However, it does not fully replicate the chronic, multifactorial nature of human OA, limiting its use in studying long-term disease progression.

A study investigating sex differences in pain perception within the CiOA mouse model highlighted that females tend to exhibit more pronounced pain sensitivity in the early stages of the disease. Behavioral assessments using methods such as the incapacitance tester, pressure application measurement, and gait analysis revealed that females showed greater weight-bearing avoidance, indicating heightened pain sensitivity. Histological analysis of joint tissues demonstrated that while both sexes exhibited cartilage damage and osteophyte formation, females correlated pain parameters with OA histological changes, with significant correlations observed in 5 out of 8 pain parameters. In contrast, only one pain-related parameter in males correlated with OA changes. These findings suggest that pain pathways in OA differ significantly between sexes, underscoring the importance of considering sex as a biological variable in OA research to better understand pain mechanisms and develop effective, sex-specific therapeutic interventions [100].

Further investigation into the CiOA rat model, which mimics the slow-progressing nature of human OA, revealed both structural and pain-related changes over time. Using contrast-enhanced microcomputed x-ray tomography (CEμCT) and gait analysis, the study demonstrated that while cartilage degeneration increased progressively from 10 to 60 days post-collagenase injection, early pain-related behavior was evident at day 10. Rats showed reduced force and time spent on the affected leg, signaling the onset of pain. This early pain was associated with inflammation, while structural damage became more apparent later, highlighting the difficulty of detecting pain prior to irreversible cartilage degeneration. The progressive nature of cartilage degeneration observed in this model more closely resembles the human condition and provides a valuable tool for studying pain management and disease progression in OA [101].

Another study focused on inflammatory and mechanical changes in the CiOA rat model further contributed to understanding the pathophysiology of OA. Collagenase injection led to dose-dependent cartilage degeneration, bone resorption, and synovitis, with more severe effects observed at higher enzyme doses. Mechanical weakening of the anterior cruciate ligament (ACL) resulted in joint destabilization, and acute inflammation, including increased cytokines and immune cell levels, was observed early on, with the systemic inflammatory response returning to baseline by day 8. While mild synovitis persisted until day 70, gait and mechanical hyperalgesia normalized after 21 days, suggesting a decoupling of pain and structural changes over time. This study emphasized the complex interplay between inflammation, mechanical instability, and pain in the CiOA model, providing insights into the underlying mechanisms of OA and the challenges of identifying pain before significant structural damage occurs [102].

### 2.6 Mechanical loading models

A mechanical loading model of OA simulates external mechanical forces, such as overuse or weight-bearing exercises, to replicate abnormal loading conditions in OA. These forces, including cyclic compression or shear, induce cartilage degradation, subchondral bone changes, and inflammation [103]. Repetitive loading mimics pathological stress, accelerating tissue degeneration and altering cellular responses like matrix remodeling, cytokine release, and chondrocyte apoptosis [104].

A study utilizing a noninvasive mechanical joint loading model in male C57BL/6 mice demonstrated that applying mechanical loading (9N or 11N) to the right knee for two weeks led to significant alterations in pain-related behaviors. Behavioral assessments revealed a marked increase in mechanical hypersensitivity and a reduction in weight-bearing capabilities two weeks after the mechanical loading. Histological analysis of the knee joints showed an increase in cartilage lesions in the loaded knees, further validating the model’s ability to induce OA-like pathology. When these mice were treated with gabapentin, diclofenac, or anti–nerve growth factor (anti-NGF), the pain phenotype was alleviated, with gabapentin proving to be the most effective analgesic. Notably, a single injection of anti-NGF provided prolonged pain relief, highlighting the potential of this model for testing new analgesic treatments for OA [103].

In addition to pain mechanisms, the role of immune cells, particularly T cells, in OA progression has gained significant attention. While the interaction between T cells and local lymph nodes in OA remains poorly understood, studies employing load-induced OA models have begun to shed light on this aspect. In a study involving C57Bl/6 female mice, mechanical loading was applied to the left tibia to simulate joint damage and induce OA-like changes. The results revealed a significant increase in T-cell populations in the lymph nodes at multiple time points, including 1-, 2-, and 6-weeks post-loading. This was accompanied by elevated expression of inflammatory cytokine markers, which are known to contribute to OA progression. These findings suggest that T cells play a crucial role in the immune response during OA and highlight the importance of studying their dynamics in rodent models of the disease [105].

## 3. Advantages and Limitations of Rodent Models in OA Research

Rodent models, particularly those involving mice and rats, are widely used in OA research due to their genetic manipulability, cost-effectiveness, and ability to recapitulate specific aspects of human OA. However, these models come with several advantages and limitations that should be carefully considered when designing OA studies.

One of the most significant advantages of rodent models is their genetic flexibility, especially in mice [106]. The availability of transgenic, knock-out, and knock-in models allows researchers to investigate the molecular mechanisms underlying OA pathogenesis with precision. These genetic tools facilitate the study of specific genes and signaling pathways implicated in cartilage degradation, synovitis, and subchondral bone remodeling, which are hallmarks of OA [107]. Additionally, rodents have a relatively short lifespan, enabling the study of disease progression and the evaluation of therapeutic interventions within a condensed timeframe [108].

Rodent models are also cost-effective and easy to maintain, making them ideal for high-throughput studies. Their small size, ease of handling, and well-characterized anatomy further contribute to their suitability for experimental studies [109]. Behavioral analyses, such as gait assessment, offer insights into OA-related pain and mobility issues. Despite differences between quadrupedal and bipedal locomotion, rodent gait abnormalities closely mirror compensatory mechanisms observed in humans with OA [110]. Moreover, non-invasive injury models in rodents enable researchers to study early OA changes without the confounding effects of surgical procedures [111]. Spontaneous OA models, such as those seen in certain mouse and guinea pig strains, replicate the slow progression of human OA, making them particularly valuable for long-term studies [112].

Additionally, experimental models of OA in rodents, such as surgical induction (e.g., anterior cruciate ligament transection) and chemically induced models (e.g., monosodium iodoacetate injection), provide reproducible and controlled methods to simulate OA-like features. These models allow researchers to examine specific disease mechanisms and evaluate therapeutic agents in a preclinical setting [113].

Despite their utility, rodent models present several limitations that must be acknowledged. The anatomical and biomechanical characteristics of rodent joints, particularly the knee, differ significantly from those of humans. Rodents, being quadrupedal, experience joint load distribution patterns that do not accurately reflect the bipedal nature of humans, hindering the study of weight-bearing stress, a key factor in human osteoarthritis (OA) [114]. OA in rodents is often induced through surgical interventions, such as destabilization of the DMM, or chemical agents like MIA. While these approaches replicate certain aspects of joint degeneration, they fail to mirror the gradual, multifactorial progression of human OA, which encompasses aging, inflammation, and metabolic factors [115]. Pain assessment in rodents is also challenging, as their subjective experience of pain cannot be directly quantified. Existing methodologies rely on behavioral proxies, such as gait analysis, which may not fully capture the complexity of OA-related pain in humans [116]. Additionally, many rodent studies predominantly utilize male subjects, despite the higher incidence of OA in women, neglecting the potential influence of hormonal and genetic factors on disease progression [117]. Additionally, while genetic models provide valuable insights, their results can be confounded by compensatory biological mechanisms unique to rodents, reducing their relevance to human conditions [118]. Finally, ethical concerns and the cost of maintaining genetically modified or aged animals pose practical limitations, particularly for long-term studies [119].

## 4. Emerging Trends in Rodent OA Research

### 4.1 Use of 3D imaging in rodent osteoarthritis models

Advancements in 3D imaging technologies have significantly enhanced the study of OA in rodent models, providing high-resolution insights into joint structures without invasive procedures. Techniques such as micro-CT and cryogenic X-ray phase-contrast imaging are pivotal in visualizing cartilage integrity, subchondral bone alterations, and cellular interactions, which are essential for early detection and monitoring of OA progression. Recent studies utilizing these methods have mapped chondrocyte hypertrophy across the endochondral interface, revealing early biomarkers of OA and subtle changes in joint composition. Notably, the role of pain in knee OA remains a critical clinical challenge, necessitating effective pharmacological interventions. However, the understanding of knee sensory innervation and molecular alterations in dorsal root ganglia (DRG) neurons is limited. Recent integrated approaches have mapped the sensory innervation of both healthy and OA-affected human and murine knees, using advanced techniques such as light sheet microscopy and spatial transcriptomics in NaV1.8;tdTomato reporter mice. These models have successfully identified key neuronal markers, such as PGP9.5+ and CGRP+, as well as endothelial markers like CD31/CD34 in osteochondral channels of human OA knees, which provide a potential target for developing therapeutics aimed at alleviating OA pain [120].

In addition, cryogenically enhanced phase contrast imaging has been applied to investigate hyaline articular cartilage in osteoarthritic (STR/Ort) and healthy (CBA) mouse knees at three different age timepoints corresponding to pre-OA, OA onset, and late progression. The research revealed that non-calcified cartilage was thicker in STR/Ort mice compared to CBA mice in several knee compartments, with significant increases in calcified and total cartilage thickness. Interestingly, trans-zonal chondrocytes, which span the tidemark, were smaller than chondrocytes in either non-calcified or calcified cartilage layers, challenging the conventional view of cartilage thinning in OA. These findings suggest a potential association between hypertrophic chondrocytes and OA progression, highlighting the complexity of cartilage remodeling in OA [121].

### 4.2 Molecular profiling in rodent OA research

Molecular profiling is an essential approach in OA research, helping to identify the genetic and biochemical pathways involved in cartilage degradation, inflammation, and repair. Techniques such as transcriptomics, proteomics, and metabolomics are increasingly used to uncover OA-associated molecular signatures. For instance, the role of miRNA 182 (miR-182) has been investigated in synovium-specific exosomes in OA. In a Sprague-Dawley rat OA model and synovial samples from OA patients, miR-182 was shown to regulate inflammation and apoptosis during OA progression. In early OA, miR-182 modulates the inflammatory response through FOXO3, whereas in advanced OA, increased miR-182 expression contributes to apoptosis signaling. These findings suggest that miR-182 is a critical player in OA progression, particularly in mediating FOXO3-driven inflammation and cell death [122].

Further investigations into sex differences in OA have revealed significant divergences in synovial transcriptomes between male and female mice, particularly in a PTOA model following anterior cruciate ligament rupture (ACLR). Male mice exhibited more severe joint damage, synovitis, pain behavior, and osteophyte formation compared to females. Notably, male mice had persistent pro-inflammatory and pro-fibrotic gene expression at 28 days post-ACLR, while females showed transcriptomic signatures indicative of inflammatory resolution. These results suggest that sustained synovial inflammation in males may be a key factor in the more severe progression of PTOA in male mice. These insights are crucial for developing gender-specific therapeutic strategies for OA (123). Such insights are crucial for developing precision-medicine approaches to OA treatment.

### 4.3 Combination models to mimic comorbid conditions

The increasing prevalence of comorbidities such as obesity and diabetes has spurred the development of combination models to better understand the multifactorial nature of OA. These models simulate the metabolic and biomechanical changes that exacerbate OA pathology. For instance, the impact of Type 1 diabetes mellitus (T1DM) on OA progression was examined in STZ-induced T1DM and control C57BL/6J mice. The results showed that T1DM mice developed mild OA phenotypes, with reduced cartilage staining and thinner cartilage compared to controls. Furthermore, RNA sequencing revealed upregulated matrix-degrading enzymes, suggesting that T1DM contributes to cartilage degradation in OA. Interestingly, in the PTOA model, T1DM mice displayed delayed disease progression with less cartilage damage and increased chondrocyte markers, highlighting the complex interplay between diabetes and OA [124].

Additionally, a DM-OA mouse model, where T1DM was induced using streptozotocin followed by surgical ACLT, revealed that diabetes accelerates OA progression through alterations in subchondral bone microarchitecture and biomechanics. In the DM-OA group, bone resorption markers were elevated, while bone formation markers were reduced, correlating with increased OA severity compared to OA and sham groups. These findings underscore the detrimental effects of diabetes on bone health and OA progression, demonstrating the need for comprehensive therapeutic strategies that address both diabetes and OA simultaneously [125].

### 4.4 Advancements in biomarkers for early detection and treatment

Biomarkers are pivotal for early OA detection and monitoring disease progression. Recent studies have identified promising molecular biomarkers, such as circulating microRNAs, glycoproteins, and cartilage-specific peptides, that reflect cartilage breakdown and inflammation. For example, the oxidative post-translational modification of collagen type II (oxPTM-CII) has been identified as an early biomarker of OA. In rat OA models, oxPTM-CII was detectable as early as 1–3 days post-surgery, before visible cartilage lesions appeared, suggesting that oxidative damage may initiate OA progression. Histological analysis confirmed that oxPTM-CII localized to the deep zone of the medial tibial cartilage, around hypertrophic chondrocytes, and co-localized with collagen type X, providing further evidence of its role in early OA pathogenesis [126].

Furthermore, biomarkers such as serum cartilage oligomeric matrix protein (COMP) and chondroitin sulfate 846 epitope (CS846) have shown diagnostic potential in OA. In a rat ACLT model, both COMP and CS846 serum levels were significantly elevated in OA rats compared to controls, with a strong correlation between these biomarkers and OA severity. Combined analysis of COMP and CS846 demonstrated a higher area under the curve (AUC) compared to individual biomarkers, suggesting that these biomarkers could be used effectively for diagnosing and monitoring OA progression [127].

These models provide a holistic understanding of the multifactorial nature of OA and inform the design of comprehensive therapeutic strategies.

## 5. Conclusion

This review has highlighted the critical role of rodent models in OA research, emphasizing their value in understanding the disease’s mechanisms, evaluating treatments, and advancing therapeutic strategies. As OA continues to be a major health concern due to aging populations and lifestyle factors, preclinical models are essential for developing effective interventions. Rodent models, including surgical, chemical, and genetic approaches, provide valuable insights into joint degeneration, inflammation, and cartilage loss, simulating key aspects of human OA. These models have been instrumental in identifying therapeutic targets, such as gene therapies, regenerative medicine, and biomaterials. However, limitations exist, particularly in translating rodent model findings to human treatments due to differences in disease progression and severity. Ethical concerns and the need for standardized protocols in experimentation also present challenges. Emerging trends, such as 3D imaging, molecular profiling, and new biomarkers, are addressing some of these issues, offering more precise insights and enhancing the predictive value of rodent models. These advancements will improve the development of targeted OA therapies. In conclusion, while rodent models remain invaluable, the continued refinement of these models, alongside innovations in imaging and molecular techniques, is key to accelerating OA therapy development and improving outcomes for patients.

## Key Points

- Rodent models are commonly used to study osteoarthritis due to their genetic similarity to humans and ease of manipulation.
- They help in understanding disease mechanisms like cartilage degradation, inflammation, and bone remodeling.
- Transgenic and knockout rodent models provide valuable insights into genetic factors contributing to osteoarthritis.
- Rodents are essential for preclinical testing of potential osteoarthritis treatments, including drugs, biologics, and regenerative therapies.
- Aging rodent models mimic age-related joint degeneration, offering insights into natural progression of osteoarthritis.
- These models enable the study of mechanical stress and joint loading in the development of osteoarthritis.
- Emerging trends in rodent osteoarthritis research focus on personalized medicine, biomarkers, and advanced imaging techniques.
- Translating findings from rodent models to humans is challenging due to differences in joint structure and disease progression.
